# Prehospital early warning scores for adults with suspected sepsis: retrospective diagnostic cohort study

**DOI:** 10.1136/emermed-2023-213315

**Published:** 2023-09-06

**Authors:** Steve Goodacre, Laura Sutton, Ben Thomas, Olivia Hawksworth, Khurram Iftikhar, Susan Croft, Gordon Fuller, Simon Waterhouse, Daniel Hind, Mike Bradburn, Michael Anthony Smyth, Gavin D Perkins, Mark Millins, Andy Rosser, Jon M Dickson, Matthew Joseph Wilson

**Affiliations:** 1 Sheffield Centre for Health and Related Research (SCHARR), The University of Sheffield, Sheffield, UK; 2 Emergency Department, Northern General Hospital, Sheffield, UK; 3 Clinical Trials Unit, University of Warwick, Coventry, UK; 4 Yorkshire Ambulance Service NHS Trust, Wakefield, UK; 5 West Midlands Ambulance Service, West Midlands, UK

**Keywords:** pre-hospital care, diagnosis, infections

## Abstract

**Background:**

Ambulance services need to identify and prioritise patients with sepsis for early hospital assessment. We aimed to determine the accuracy of early warning scores alongside paramedic diagnostic impression to identify sepsis that required urgent treatment.

**Methods:**

We undertook a retrospective diagnostic cohort study involving adult emergency medical cases transported to Sheffield Teaching Hospitals ED by Yorkshire Ambulance Service in 2019. We used routine ambulance service data to calculate 21 early warning scores and categorise paramedic diagnostic impressions as sepsis, infection, non-specific presentation or other presentation. We linked cases to hospital records and identified those meeting the sepsis-3 definition who received urgent hospital treatment for sepsis (reference standard). Analysis determined the accuracy of strategies that combined early warning scores at varying thresholds for positivity with paramedic diagnostic impression.

**Results:**

We linked 12 870/24 955 (51.6%) cases and identified 348/12 870 (2.7%) with a positive reference standard. None of the strategies provided sensitivity greater than 0.80 with positive predictive value greater than 0.15. The area under the receiver operating characteristic curve for the National Early Warning Score, version 2 (NEWS2) applied to patients with a diagnostic impression of sepsis or infection was 0.756 (95% CI 0.729, 0.783). No other early warning score provided clearly superior accuracy to NEWS2. Paramedic impression of sepsis or infection had sensitivity of 0.572 (0.519, 0.623) and positive predictive value of 0.156 (0.137, 0.176). NEWS2 thresholds of >4, >6 and >8 applied to patients with a diagnostic impression of sepsis or infection, respectively, provided sensitivities and positive predictive values of 0.522 (0.469, 0.574) and 0.216 (0.189, 0.245), 0.447 (0.395, 0.499) and 0.274 (0.239, 0.313), and 0.314 (0.268, 0.365) and 0.333 (0.284, 0.386).

**Conclusion:**

No strategy is ideal but using NEWS2 alongside paramedic diagnostic impression of infection or sepsis could identify one-third to half of sepsis cases without prioritising unmanageable numbers. No other score provided clearly superior accuracy to NEWS2.

**Trial registration number:**

researchregistry5268, https://www.researchregistry.com/browse-the-registry%23home/registrationdetails/5de7bbd97ca5b50015041c33/

**WHAT IS ALREADY KNOWN ON THIS TOPIC**

- Guidelines for sepsis recommend urgent treatment within 1 hour for people with suspected sepsis who are at highest risk. Ambulance services can use early warning scores alongside paramedic diagnostic impression to identify and prioritise people with suspected sepsis.

**WHAT THIS STUDY ADDS**

- This retrospective diagnostic cohort study of 12 870 patients showed that no combination of early warning score alongside diagnostic impression provides sensitivity greater than 0.80 with positive predictive value greater than 0.15. Using the National Early Warning Score, version 2 (NEWS2) at thresholds of >4 to >8 in patients with a diagnostic impression of infection or sepsis could identify one-third to half of sepsis cases without prioritising unmanageable numbers. No alternative early warning score provided clearly superior accuracy to NEWS2.

**HOW THIS STUDY MIGHT AFFECT RESEARCH, PRACTICE OR POLICY**

- Ambulance services and hospitals can use the estimates of NEWS2 sensitivity and positive predictive value to identify an appropriate NEWS2 threshold score to guide the use of prealerts for patients with suspected sepsis.

## Introduction

Sepsis is a life-threatening response to a severe infection, which can lead to tissue damage, organ failure and death.^1^ Guidelines for sepsis highlight the importance of early recognition and treatment, with treatment recommended within 1 hour of presentation for those at highest risk.^1–4^ The emergency care system can only achieve this if sepsis is recognised and prioritised. This may involve ambulance services prealerting the ED that they are transporting a patient with suspected sepsis. However, prioritising too many patients with suspected sepsis may delay assessment of other urgent cases or may result in a lack of meaningful prioritisation.

Ambulance services can use prehospital early warning scores to identify people with a high risk of sepsis.^5^ Early warning scores use clinical observations to determine a score, with a higher score indicating a higher risk of adverse outcome. They may be generic (applicable to a range of conditions) or specific to sepsis. Clinicians need to determine a threshold value of the score for decision-making that balances the risks of missing sepsis against prioritising too many patients. Sepsis may present with non-specific symptoms,^1^ so clinicians need to decide whether to suspect sepsis and apply an early warning score to all medical cases, non-specific presentations, suspected infection or just suspected sepsis.

Systematic reviews have identified many potential prehospital early warning scores for sepsis but supporting evidence has substantial weaknesses and reports inconsistent findings.^6–8^ This may be explained by differences in study populations, reference standard definitions, the threshold score used or whether the score was applied to all medical cases or just those with evidence of infection.^5^

Evaluating the accuracy of an early warning score or diagnostic assessment for sepsis involves determining the sensitivity (to reflect the risk of missing sepsis) and the specificity (to reflect the risk of prioritising cases without sepsis). A score with apparently high specificity may prioritise an unmanageable number of cases if the prevalence of sepsis is low, such as when the score is applied to all medical cases. Furthermore, specificity (the proportion of patients without sepsis who have a score below the threshold) may be difficult to interpret in clinical practice. We therefore use positive predictive value (the proportion of patients with a score above the threshold who have sepsis) rather than specificity to interpret the risk of prioritising too many patients.

We aimed to determine the accuracy of prehospital early warning scores, used alongside paramedic diagnostic impression, for identifying sepsis requiring urgent treatment in adult medical cases transported to hospital by emergency ambulance.

## Methods

This study is the main component of the Prehospital Early Warning Scores for Sepsis study. Full details of the project will be reported in the National Institute for Health Research library.^9^ We planned to undertake a retrospective observational cohort study across two ambulance services and four hospitals using the UK NHS Data Access Request Service from NHS Digital to link ambulance service to hospital data. However, NHS Digital was unable to provide this service, so we implemented an alternative process using NHS numbers (a unique number for each NHS patient) to link Yorkshire Ambulance Service data to Sheffield Teaching Hospitals data.

We used routine ambulance service data to identify all adult emergency medical cases transported to the Sheffield Teaching Hospitals ED from 1 January to 31 December 2019. We excluded cases with injury, mental health problems, cardiac arrest or direct transfer to specialist services (including maternity, cardiac or stroke services). We also excluded cases with no NHS number and patients who had opted out of allowing use of their data for research. Individuals can inform NHS Digital or their general practice that they wish to opt out of having their NHS data used for research and planning purposes. Yorkshire Ambulance Service checked cases against the national data opt-out service and removed records from the data set if they were identified as belonging to individuals who have opted out.

We evaluated any early warning score that prehospital professionals could use and that we could calculate from the ambulance service electronic patient report form (ePRF). We included dichotomous scores (ie, rules) that simply categorise into high and low-risk groups, but for simplicity refer collectively to early warning scores. We searched the Embase, CINAHL, PubMed, ClinicalTrials.gov, the ISRCTN registry and Research Registry for relevant studies and selected 21 scores for evaluation.^3 10–29^ Online supplemental table 1 outlines the scores and compares their constituent variables. The scores used combinations of age, temperature, HR, RR, peripheral oxygen saturation, conscious level and BP, along with a small number of other variables. During the study period, Yorkshire Ambulance Service used an electronic patient record that calculated the National Early Warning Score, version 2 (NEWS2)^10^ from constituent variables so paramedics would have been aware of this score.

10.1136/emermed-2023-213315.supp2 Supplementary data

We calculated each score for each case using ePRF data. We used the first recorded measurement for each variable. If the variable was not recorded in the first set of observations, then the first recorded measurement was used from a subsequent set of observations. We inferred conscious level or ACVPU (alert, confused, voice, pain, unresponsive) from the GCS, assuming 15 equals alert, 14 equals confused, 12–13 equal voice, 9–11 equal pain and 3–8 equal unresponsive. We modified scores that included variables that would not be available in routine practice or were not recorded on the ePRF. For example, we removed lactate, oliguria and recent chemotherapy from the UK Sepsis Trust red flag criteria.^3^ Online supplemental appendix 1 provides details of how each score is calculated, any modifications or assumptions in calculating the score from routine data and the threshold for decision-making.

10.1136/emermed-2023-213315.supp1 Supplementary data

The ePRF recorded a paramedic diagnostic impression from a list of options. We categorised the options as sepsis, infection (excluding sepsis), non-specific diagnostic impression in which sepsis could be suspected or other diagnostic impression in which sepsis would not usually be suspected (see online supplemental appendix 2 for details). We then applied each early warning score alongside diagnostic impression as follows:

1. Score applied to cases with impression of sepsis. Cases with impression of infection, non-specific or other were categorised as score negative.
2. Score applied to cases with impression of sepsis or infection. Cases with impression of non-specific or other were categorised as score negative.
3. Score applied to cases with impression of sepsis, infection or non-specific. Cases with impression of other were categorised as score negative.
4. Score applied to all cases regardless of diagnostic impression.

We defined the reference standard (sepsis requiring urgent treatment) as being positive if the patient met the sepsis-3 definition of sepsis and received treatment for sepsis within 4 hours of initial assessment at hospital.^30^ We planned a secondary analysis using just the sepsis-3 definition as the reference standard but 95% of cases meeting the sepsis-3 definition received urgent treatment, so the results of the secondary analysis matched the primary analysis. We therefore only report the primary analysis.

We used routine hospital data to select those with a primary or secondary International Classification of Diseases 10 admission code or cause of death compatible with sepsis, or an ED code for sepsis. Research nurses briefly reviewed the ED records of these cases and selected patients for expert review if they had any diagnosis or treatment for sepsis recorded in the ED notes or sepsis as an admission diagnosis on the hospital discharge summary.

Two experts independently reviewed hospital records for the selected patients and determined whether there was: (1) evidence of infection and life-threatening organ dysfunction (according to the sepsis-3 definition^30^) within 4 hours of initial assessment; and (2) treatment for sepsis given within 4 hours. Evidence of infection could include microbiology reports identifying organisms, radiology reports identifying infective changes or other markers strongly suggesting infection. Organ dysfunction was defined as a Sequential (sepsis-related) Organ Failure Assessment (SOFA) score of 2 or more points worse than normal. We estimated the SOFA score using the ED observations chart and first blood results after admission. In accordance with the sepsis-3 definition,^30^ we assumed the normal SOFA score would be 0 unless there was evidence in the hospital records to suggest otherwise. Treatment for sepsis was based on relevant guidelines^1 2^ and typically involved intravenous antibiotic therapy. One of the experts also estimated the Clinical Frailty Score using information in the hospital records.^31^

If the two reviewers disagreed on the overall sepsis-3 judgement or whether urgent treatment for sepsis was given, then a consensus decision was reached through discussion. Disagreements over an element of the sepsis-3 definition (evidence of infection or change in SOFA score) were left unresolved if they did not affect the overall judgement.

We used the patient as the unit of analysis and only included the first eligible episode per patient. Kappa scores were calculated to determine the agreement between reference standard adjudicators. We constructed receiver operating characteristic (ROC) curves to evaluate sensitivity and specificity over the range of each score. We calculated the area under the ROC curve and sensitivities, specificities and positive and negative predictive values at key cut-points, each with a 95% CI.

We anticipated a low prevalence of reference standard positive cases, based on data from Smyth *et al*,^27^ so we based the sample size on identifying at least 200 reference standard positive cases. Collins *et al* 32 recommend basing external validation studies on a minimum of 100–200 events.^32^ Our sample size would allow us to estimate the sensitivity of an early warning score with an SE of 2.1% assuming sensitivity of 90%, and the area under the ROC curve with an SE of 2% assuming an area under the ROC curve of at least 0.75.^33^

Clinical experts in the research team reviewed ED attendance data and determined that a positive predictive value of 0.15 or lower would result in too many positive cases for meaningful prioritisation and that sensitivity exceeding 0.8 would be considered good.

### Patient and public involvement

The Sheffield Emergency Care Forum (SECF) is a public representative group interested in emergency care research.^34^ Two members of SECF joined the project management group and helped develop and deliver the project. Public representatives supported the use of patient data without consent and reviewed the early warning scores to determine patient and public acceptability, resulting in one score being modified to remove care home residence as a variable. Patients were not involved in the recruitment to and conduct of the study. We are unable to disseminate the findings to study participants directly.

## Results

**Figure 1:**
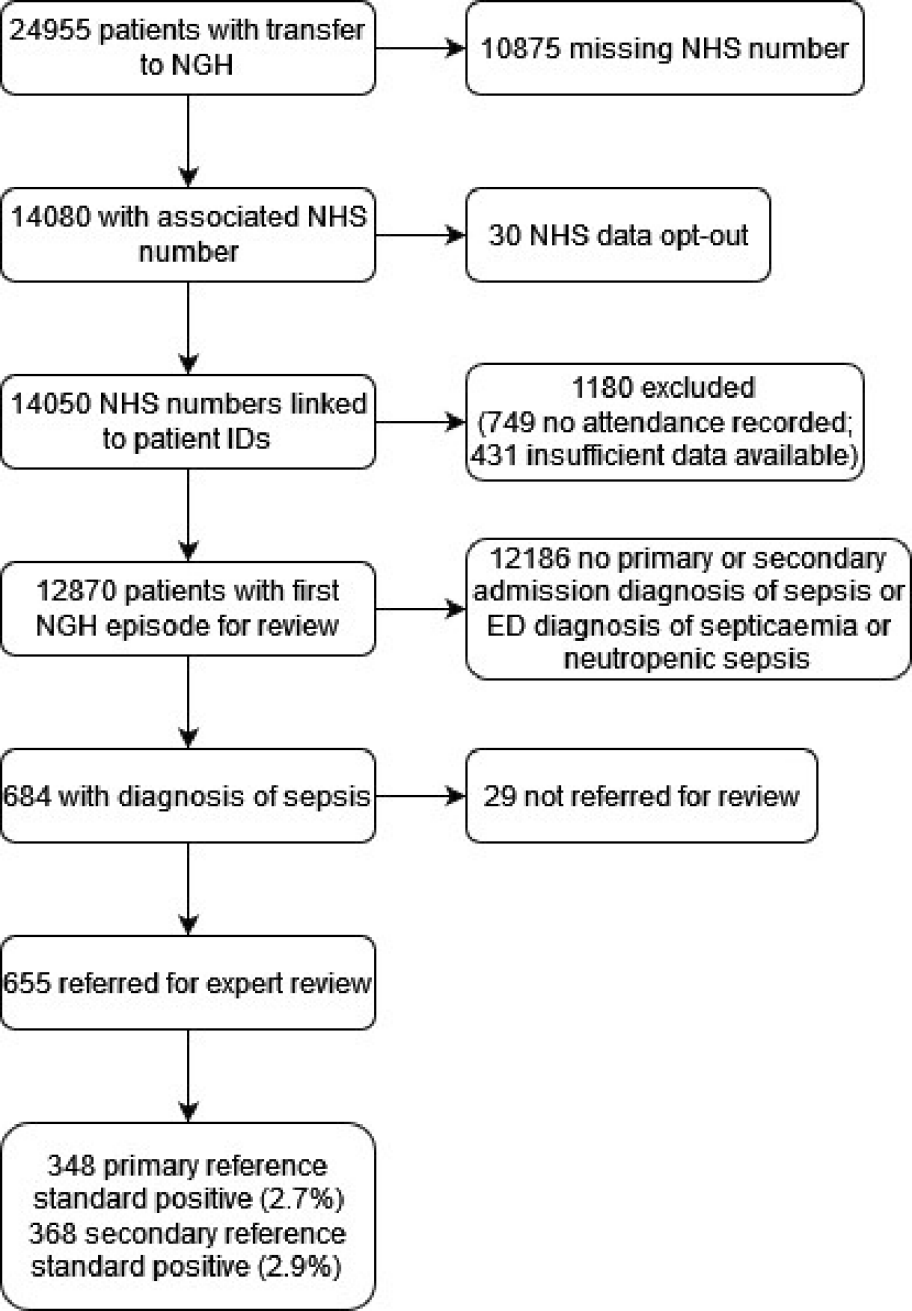
Participant flow through the study. NGH, Northern General Hospital.

Figure 1 shows the flow of eligible cases. We identified 24 955 cases transported to Sheffield Teaching Hospitals ED in 2019, of whom 14 050 (56.3%) had NHS numbers and no opt-out. Table 1 shows the characteristics of the 14 050 patients and compares them to those unavailable for linkage. Included patients were markedly older (median age 71 vs 55 years) and more likely to be female (54.7% vs 53.0%) and white ethnicity (95.7% vs 91.8%). We linked 12 870/14 050 cases (91.6%) with a hospital attendance or admission, which comprised the study cohort.

**Table 1:** Characteristics of patients available for linkage with hospital data

	Not linked (n=10 905)	Linked (n=14 050)	Total (n=24 955)
Age (years)			
Mean (SD)	55.2 (23.3)	65.3 (21.2)	60.9 (22.7)
Median (IQR)	55.0 (34.0, 76.0)	71.0 (51.0, 82.0)	65.0 (42.0, 80.0)
Range	16.0–102.0	16.0–105.0	16.0–105.0
Sex			
Missing	0	22	22
Female	5484 (50.3%)	7672 (54.7%)	13 156 (52.8%)
Male	5421 (49.7%)	6356 (45.3%)	11 777 (47.2%)
Ethnicity			
Missing	5290	6880	12 170
White	5153 (91.8%)	6860 (95.7%)	12 013 (94.0%)
Asian	136 (2.4%)	122 (1.7%)	258 (2.0%)
Black	73 (1.3%)	55 (0.8%)	128 (1.0%)
Mixed	49 (0.9%)	32 (0.4%)	81 (0.6%)
Other	204 (3.6%)	101 (1.4%)	305 (2.4%)
ACVPU			
Missing	0	0	0
Alert	9754 (89.4%)	13 232 (94.2%)	22 986 (92.1%)
Confusion	341 (3.1%)	387 (2.8%)	728 (2.9%)
Voice	386 (3.5%)	257 (1.8%)	643 (2.6%)
Pain	192 (1.8%)	107 (0.8%)	299 (1.2%)
Unresponsive	232 (2.1%)	67 (0.5%)	299 (1.2%)
GCS			
Mean (SD)	14.4 (2.0)	14.7 (1.2)	14.5 (1.6)
Median (IQR)	15.0 (15.0, 15.0)	15.0 (15.0, 15.0)	15.0 (15.0, 15.0)
Range	3.0–15.0	3.0–15.0	3.0–15.0
Diastolic BP (mm Hg)			
Mean (SD)	83.1 (17.5)	82.1 (17.2)	82.6 (17.4)
Median (IQR)	83.0 (72.0, 94.0)	82.0 (71.0, 93.0)	82.0 (71.0, 93.0)
Range	0.0–190.0	5.0–195.0	0.0–195.0
Systolic BP (mm Hg)			
Mean (SD)	139.0 (26.5)	142.1 (27.4)	140.8 (27.1)
Median (IQR)	138.0 (122.0, 153.0)	140.0 (124.0, 158.0)	139.0 (123.0, 156.0)
Range	53.0–257.0	43.0–285.0	43.0–285.0
HR (beats/min)			
Mean (SD)	89.5 (22.8)	88.7 (21.9)	89.1 (22.3)
Median (IQR)	87.0 (74.0, 103.0)	86.0 (73.0, 102.0)	86.0 (74.0, 102.0)
Range	0.0–218.0	0.0–216.0	0.0–218.0
Oxygen saturation (%)			
Mean (SD)	96.0 (4.9)	95.6 (4.9)	95.8 (4.9)
Median (IQR)	97.0 (95.0, 98.0)	97.0 (95.0, 98.0)	97.0 (95.0, 98.0)
Range	18.0–100.0	10.0–100.0	10.0–100.0
Supplemental oxygen			
Missing	18	27	45
No	10 345 (95.0%)	13 252 (94.5%)	23 597 (94.7%)
Yes	542 (5.0%)	771 (5.5%)	1313 (5.3%)
Respiration (breath/min)			
Mean (SD)	19.7 (6.0)	20.5 (6.1)	20.1 (6.0)
Median (IQR)	18.0 (16.0, 20.0)	18.0 (16.0, 22.0)	18.0 (16.0, 22.0)
Range	0.0–93.0	0.0–91.0	0.0–93.0
Temperature (°C)			
Mean (SD)	36.8 (1.0)	37.0 (1.0)	36.9 (1.0)
Median (IQR)	36.8 (36.2, 37.3)	36.9 (36.4, 37.4)	36.8 (36.4, 37.4)
Range	26.0–41.3	27.1–41.8	26.0–41.8
Glucose (mmol/L)			
Mean (SD)	7.1 (3.2)	7.4 (3.4)	7.2 (3.3)
Median (IQR)	6.2 (5.4, 7.6)	6.4 (5.5, 8.0)	6.3 (5.5, 7.8)
Range	0.5–36.6	0.9–49.0	0.5–49.0
Prealerted			
No	10 307 (94.5%)	13 419 (95.5%)	23 726 (95.1%)
Yes	598 (5.5%)	631 (4.5%)	1229 (4.9%)
Impression			
1—Sepsis	222 (2.0%)	407 (2.9%)	629 (2.5%)
2—Infection	471 (4.3%)	912 (6.5%)	1383 (5.5%)
3—Non-specific	3494 (32.0%)	4962 (35.3%)	8456 (33.9%)
4—Other	6718 (61.6%)	7769 (55.3%)	14 487 (58.1%)

There were 684/12 870 episodes with an admission or ED coding for sepsis. The research nurses referred 655/684 (95.8%) for expert review. The experts judged that 368/655 (56.2%) episodes met the sepsis-3 definition and 348/368 (94.6%) of these received urgent treatment for sepsis. Therefore, 348/12 870 (2.7%) met the reference standard definition. Online supplemental table 2 shows the agreement between the reference standard adjudicators. Agreement was moderate (kappa=0.62) for evidence of infection but disagreements tended to occur in cases that did not meet the SOFA score criterion, so overall judgement on the sepsis-3 definition was good (kappa=0.89), as was agreement for whether urgent treatment was given (kappa=0.87).

There was radiological evidence of infection in 175/348 (50.1%) cases, microbiological evidence in 171 (49.0%) and other clinical evidence in 328 (94.0%). The sites of suspected infection were chest (155, 44.4%), urine (78, 22.3%), biliary (43, 12.3%), abdominal (16, 4.6%), skin (25, 7.2%), other (6, 1.7%)and unknown (26, 7.4%). Mean Clinical Frailty Score was 5.6 (median 6.0, range 2.0–9.0) and mean SOFA score was 3.9 (median 3.0, range 2.0–14.0). Some 28 (8.0%) were admitted to critical care and 261 (74.8%) survived to hospital discharge or 30 days after attendance, whichever was sooner.

Paramedic diagnostic impression of sepsis had sensitivity (95% CI) of 0.328 (0.28, 0.379) and positive predictive value of 0.285 (0.243, 0.331); infection or sepsis had sensitivity of 0.572 (0.519, 0.623) and positive predictive value of 0.156 (0.137, 0.176); and non-specific, infection or sepsis had sensitivity of 0.897 (0.86, 0.924) and positive predictive value of 0.053 (0.048, 0.059). Online supplemental table 3 shows the full details.

**Figure 2:**
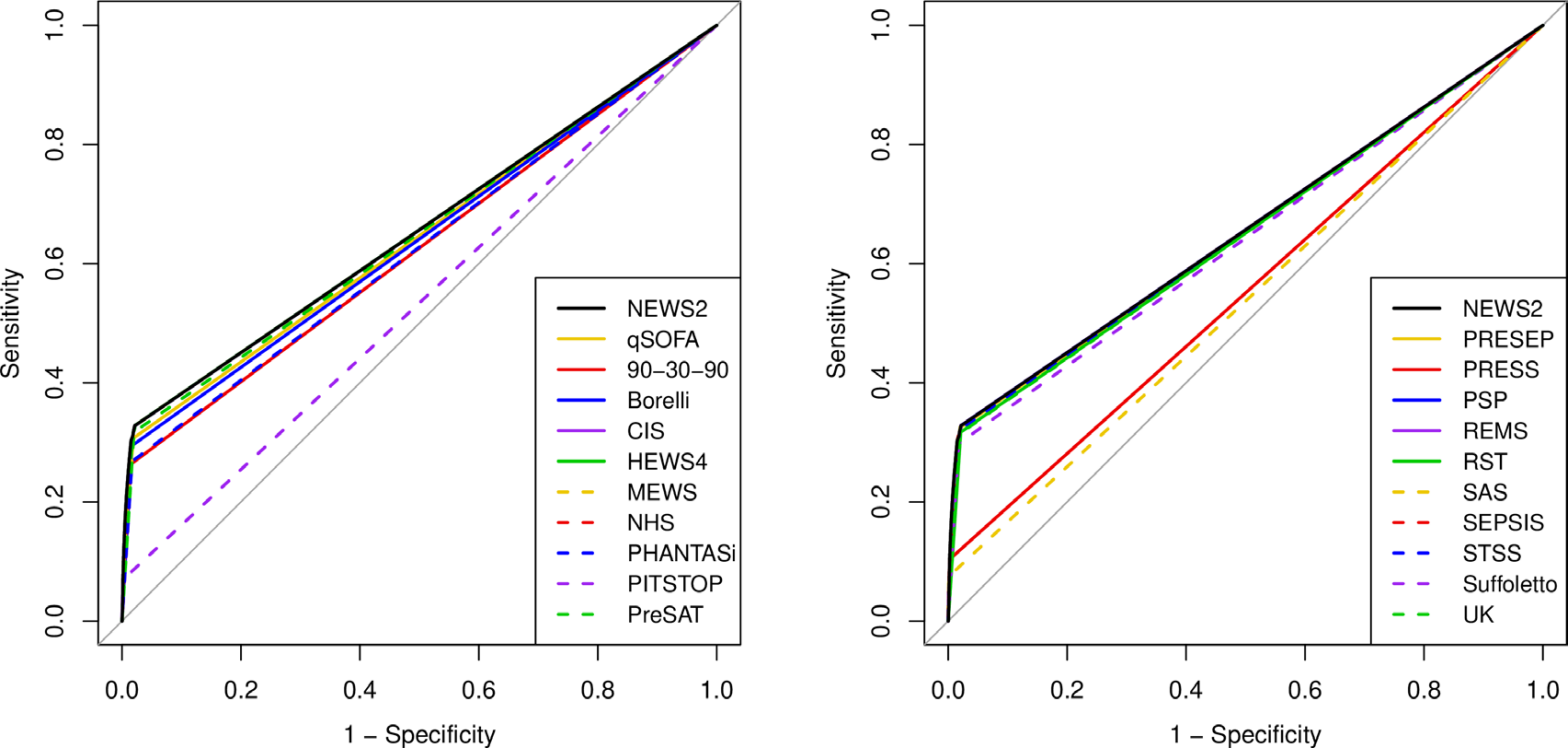
Receiver operating characteristic (ROC) curves for early warning scores applied to diagnostic impression of sepsis. CIS, Critical Illness Score; HEWS, Hamilton Early Warning Score; MEWS, Modified Early Warning Score; NEWS2, National Early Warning Score, version 2; PHANTASi, Prehospital Antibiotics Against Sepsis; PITSTOP, Paramedic Initiated Treatment of Sepsis Targeting Out-of-hospital Patients; PreSAT, Prehospital Sepsis Assessment Tool; PRESEP, Prehospital Early Sepsis Detection; PRESS, Prehospital Severe Sepsis; PSP, Prehospital Sepsis Project; qSOFA, quick Sequential Organ Failure Assessment; REMS, Rapid Emergency Medicine Score; RST, Robson Screening Tool; SEPSIS, Screening to Enhance Prehospital Identification of Sepsis; STSS, Simple Triage Scoring System.

**Figure 3:**
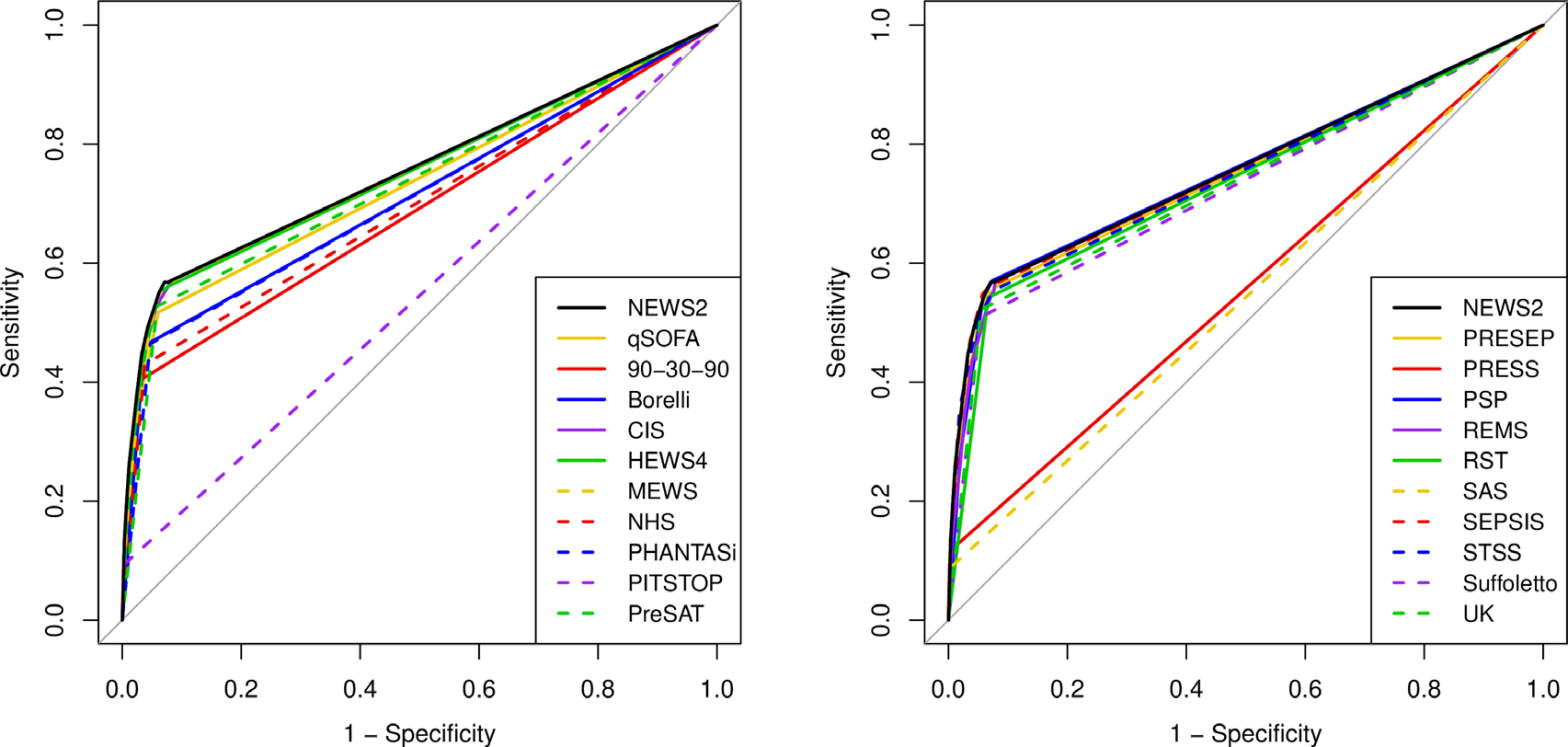
Receiver operating characteristic (ROC) curves for early warning scores applied to diagnostic impression of sepsis or infection. CIS, Critical Illness Score; HEWS, Hamilton Early Warning Score; MEWS, Modified Early Warning Score; NEWS2, National Early Warning Score, version 2; PHANTASi, Prehospital Antibiotics Against Sepsis; PITSTOP, Paramedic Initiated Treatment of Sepsis Targeting Out-of-hospital Patients; PreSAT, Prehospital Sepsis Assessment Tool; PRESEP, Prehospital Early Sepsis Detection; PRESS, Prehospital Severe Sepsis; PSP, Prehospital Sepsis Project; qSOFA, quick Sequential Organ Failure Assessment; REMS, Rapid Emergency Medicine Score; RST, Robson Screening Tool; SEPSIS, Screening to Enhance Prehospital Identification of Sepsis; STSS, Simple Triage Scoring System.

**Figure 4:**
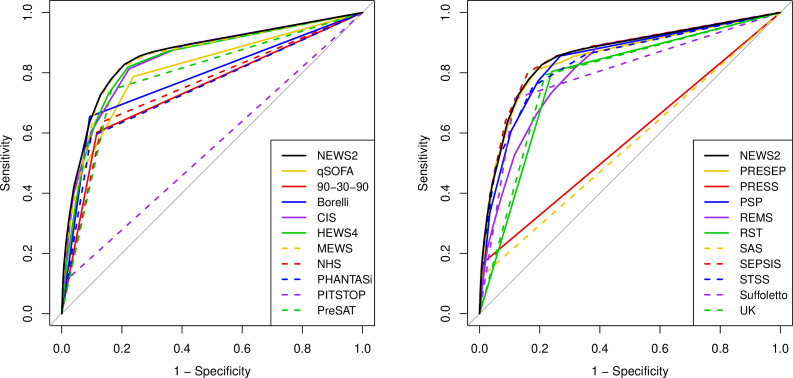
Receiver operating characteristic (ROC) curves for early warning scores applied to diagnostic impression of sepsis, infection or non-specific presentation. CIS, Critical Illness Score; HEWS, Hamilton Early Warning Score; MEWS, Modified Early Warning Score; NEWS2, National Early Warning Score, version 2; PHANTASi, Prehospital Antibiotics Against Sepsis; PITSTOP, Paramedic Initiated Treatment of Sepsis Targeting Out-of-hospital Patients; PreSAT, Prehospital Sepsis Assessment Tool; PRESEP, Prehospital Early Sepsis Detection; PRESS, Prehospital Severe Sepsis; PSP, Prehospital Sepsis Project; qSOFA, quick Sequential Organ Failure Assessment; REMS, Rapid Emergency Medicine Score; RST, Robson Screening Tool; SEPSIS, Screening to Enhance Prehospital Identification of Sepsis; STSS, Simple Triage Scoring System.

**Figure 5:**
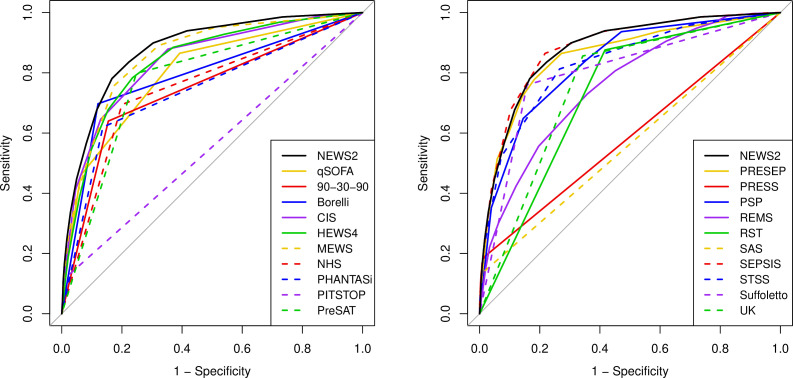
Receiver operating characteristic (ROC) curves for early warning score applied to all diagnostic impressions. CIS, Critical Illness Score; HEWS, Hamilton Early Warning Score; MEWS, Modified Early Warning Score; NEWS2, National Early Warning Score, version 2; PHANTASi, Prehospital Antibiotics Against Sepsis; PITSTOP, Paramedic Initiated Treatment of Sepsis Targeting Out-of-hospital Patients; PreSAT, Prehospital Sepsis Assessment Tool; PRESEP, Prehospital Early Sepsis Detection; PRESS, Prehospital Severe Sepsis; PSP, Prehospital Sepsis Project; qSOFA, quick Sequential Organ Failure Assessment; REMS, Rapid Emergency Medicine Score; RST, Robson Screening Tool; SEPSIS, Screening to Enhance Prehospital Identification of Sepsis; STSS, Simple Triage Scoring System.

Figures 2–5 show the ROC curves for each score alongside diagnostic impression of sepsis, infection, non-specific and all cases. Online supplemental table 4 reports the areas under each ROC curve and online supplemental tables 5–12 show the accuracy parameters behind the ROC curves. The area under the ROC curve is greater when the scores are used less selectively with paramedic diagnostic impression. However, the accuracy parameters in the online supplemental tables show that the positive predictive value is low (<0.15) if specificity is below 0.9. The area under the ROC curve is therefore a poor reflection of accuracy at the thresholds that yield acceptable positive predictive value (ie, specificity >0.9). Figures 2–5 show that none of the alternative scores had superior accuracy to NEWS2. The possible exception is the Screening to Enhance Prehospital Identification of Sepsis (SEPSIS) score that has a higher area under the ROC curve when applied to non-specific or all cases, but has similar accuracy to NEWS2 at thresholds that provide specificity greater than 0.9.

Table 2 shows the accuracy parameters (reproduced from online supplemental tables 5–12) for early warning scores at specified thresholds, selected on the basis of their use in sepsis guidelines^3 4 17 30^ in patients with an impression of infection or sepsis. NEWS2>4, NEWS2>6 and quick SOFA (qSOFA)>1 provide a range of options with varying sensitivity and positive predictive value. qSOFA>1 provides similar accuracy to NEWS2>8 (also included in the table). The modified NHS prealert criteria^17^ provide slightly inferior accuracy to NEWS2>6. The modified UK Sepsis Trust criteria^3^ provide similar accuracy to NEWS2>4.

**Table 2:** Accuracy of selected early warning scores alongside paramedic impression of sepsis or infection for identifying sepsis receiving urgent treatment

Early warning score	Sensitivity (95% CI)	Specificity (95% CI)	PPV (95% CI)	NPV (95% CI)
Paramedic impression alone	0.572 (0.519, 0.623)	0.914 (0.909, 0.919)	0.156 (0.137, 0.176)	0.987 (0.985, 0.989)
NEWS2>4	0.522 (0.469, 0.574)	0.947 (0.943, 0.951)	0.216 (0.189, 0.245)	0.986 (0.984, 0.988)
NEWS2>6	0.447 (0.395, 0.499)	0.967 (0.964, 0.97)	0.274 (0.239, 0.313)	0.984 (0.982, 0.986)
NEWS2>8	0.314 (0.268, 0.365)	0.983 (0.98, 0.985)	0.333 (0.284, 0.386)	0.981 (0.978, 0.983)
qSOFA>1	0.305 (0.259, 0.355)	0.985 (0.982, 0.987)	0.356 (0.304, 0.412)	0.981 (0.978, 0.983)
NHS prealert	0.429 (0.378, 0.482)	0.962 (0.959, 0.966)	0.24 (0.208, 0.275)	0.984 (0.981, 0.986)
UK Sepsis Trust	0.522 (0.469, 0.574)	0.945 (0.941, 0.949)	0.209 (0.183, 0.237)	0.986 (0.984, 0.988)

NEWS2, National Early Warning Score, version 2; NPV, negative predictive value; PPV, positive predictive value; qSOFA, quick Sequential Organ Failure Assessment.

## Discussion

We found that no combination of early warning score alongside paramedic diagnostic impression provided sensitivity greater than 0.8 and positive predictive value greater than 0.15 for sepsis. The appropriate trade-off between sensitivity and positive predictive value will depend on the consequences of prioritisation. However, prioritising more than five people for each case of sepsis (which would be the consequence of using a strategy with positive predictive value of 0.15 or lower) risks overstretching ED capacity and a loss of meaningful prioritisation.

No score had superior accuracy to NEWS2. The only possible exception was the SEPSIS score^27^ when thresholds were used that optimised sensitivity at the expense of low positive predictive value. NEWS2 is widely used in the UK NHS, so any alternative score would need to demonstrate clear superiority to justify the additional training and documentation required in this setting. Using NEWS2 at thresholds of >4 and >6 to prioritise patients with suspected infection, as recommended by the Academy of Medical Royal Colleges clinical decision support framework,^4^ would provide sensitivities of 0.522 and 0.447, respectively, and positive predictive values of 0.216 and 0.274. To prioritise fewer patients, NEWS2 could be used with a threshold of >8, which would provide similar sensitivity and positive predictive value (0.314 and 0.333) to using qSOFA with a threshold of >1 (0.305 and 0.356). Using NEWS2 alongside paramedic diagnostic impression improves positive predictive value at the expense of sensitivity, compared with paramedic diagnostic impression alone.

We recently searched for studies validating the accuracy of early warning scores for suspected sepsis in a prehospital population and identified 13 studies evaluating the scores included in this study.^5^ There was substantial variation in the reported results, with no consistent evidence that any score was superior to the others. Variations in study populations, outcomes and the thresholds used make comparisons difficult. A systematic review of hospital studies found that at established thresholds NEWS tended to have higher sensitivity while qSOFA tended to have higher specificity.^35^ Our study suggests that this difference reflects the chosen threshold. The sensitivity and specificity of NEWS2 at a higher threshold than usually recommended (>8) are similar to the sensitivity and specificity of qSOFA>1.

Our findings are similar to other studies evaluating multiple scores in a large cohort. Lane *et al* found that no single strategy had high sensitivity and specificity for classifying sepsis, but the Critical Illness Prediction score, NEWS and qSOFA showed good prediction for sepsis.^36^ Smyth *et al* identified three strategies offering an acceptable balance between sensitivity and positive predictive value: SEPSIS>2, Systemic Inflammatory Response Syndrome (SIRS) criteria >1 and NEWS>4.^27^ These studies did not identify any early warning score with clearly superior accuracy to NEWS2.

Key strengths of our study include the large sample size including sufficient cases with sepsis to estimate sensitivity with reasonable precision. The reference standard was based on an internationally recognised definition of sepsis that was adjudicated by two independent clinicians with acceptable interobserver agreement. The main limitation is that we were only able to link around half the eligible cases with hospital records. Those linked tended to be much older, possibly reflecting more frequent contact with health services. Sepsis is associated with age and comorbidity, but our findings may not be generalisable to younger patients with little comorbidity. The single-centre design limits the generalisability of the findings. The predominantly white ethnicity of our population may limit generalisability to patients of other ethnicities. We collected data over a year to mitigate the effects of seasonality and used data from 2019 as we felt that this was a typical year in terms of the prevalence of respiratory pathogens (if such a thing exists), but rates of presentations requiring prioritisation may show marked seasonality and variation according to the prevalence of respiratory pathogens. We may have misclassified cases as reference standard negative if they had sepsis but the ED or hospital discharge codes did not include sepsis.

Other limitations relate to the sepsis-3 definition.^30^ While adjudicating the reference standard we noticed that the change in SOFA score often reflected the local effects of infection (eg, respiratory failure in pneumonia or raised bilirubin in biliary infection) or an exacerbation of underlying comorbidity, rather than organ failure likely to reflect a dysregulated host response to infection. The sepsis-3 definition was based on evidence that the SOFA score predicts mortality,^11^ but this may not translate into potential to benefit from treatment.^37 38^ Our reference standard may therefore include many patients who do not have a dysregulated response to infection and are unlikely to benefit from early treatment. We tried to address this issue by including receipt of urgent treatment for sepsis in our definition, but 95% of presentations received early treatment for sepsis.

Paramedic awareness of the NEWS2 score may have influenced their assessment of diagnostic impression, particularly in terms of differentiating sepsis from other infections. This may mean that paramedic diagnostic impression and NEWS2 scores are correlated to a degree. Use of NEWS2 in the ED may have prompted greater investigation for infection in patients with a higher NEWS2 score. However, NEWS2 scores were not routinely recorded in the hospital records used in reference standard assessment, so the reference standard adjudicators were not aware of the patient’s NEWS2 (or any other) score.

The implications of our findings are that any combination of diagnostic impression and early warning score is likely to result in too many cases being prioritised or cases of sepsis being missed. EDs must therefore either ensure capacity to handle large numbers of cases being prioritised or ensure that missed cases do not suffer excessive delays. Ambulance services could use NEWS2 in patients with evidence of infection at thresholds of >4, >6 or >8, depending on the capacity of EDs to handle prioritised cases or avoid excessive delay for missed cases.

Future research is required to improve prehospital identification of sepsis but new scores based on currently measured physiological parameters are unlikely to improve on NEWS2. Prehospital biomarkers could improve early warning scores but future research needs to address the limitations of the sepsis-3 definition. Until we are able to measure the dysregulated host response that characterises sepsis, we will risk developing methods that identify patients with infection and organ failure, but do not have a dysregulated host response.

In summary, we found no ideal strategy but using NEWS2 alongside paramedic diagnostic impression of infection or sepsis could identify one-third to half of sepsis cases without prioritising unmanageable numbers.

## Data Availability

Anonymised data are available from the corresponding author upon reasonable request (contact s.goodacre@sheffield.ac.uk).
