# Supplementary material for: Prehospital early warning scores for adults with suspected sepsis: retrospective diagnostic cohort study
Source: Emerg Med J. 2023 Sep 6;40(11):768–76. doi: 10.1136/emermed-2023-213315 (PMC10646863; doi:10.1136/emermed-2023-213315)
Supplement: Supplementary data [file emermed-2023-213315supp002.pdf]

Supplementary tables

Supplementary Table 1: Early warning scores and constituent variables

Early Warning Score	Age	Temperature	Heart rate	Respiratory rate	Oxygen saturation	Conscious level	Blood pressure	Other
NEWS2 [10]		X	X	X	X	X	X	Inspired oxygen
qSOFA [11]				X		X	X	
90-30-90 [12]				X	X		X	
Borelli [13]		X	X	X	X	X	X	Suspected infection
CIS [14]	X		X	X	X	X	X	
HEWS [15]		X	X	X	X	X	X	Inspired oxygen
MEWS [16]		X	X	X		X	X	
NHS pre-alert [17]			X	X	X	X	X	
PHANTASi [18]		X	X	X				
PITSTOP [19]		X					X	Paramedic suspicion of infection
PreSAT [20]		X	X	X			X	
PRESEP [21]		X	X	X	X		X	
PRESS [22]	X	X			X		X	Dispatch chief complaint of sick person; nursing home resident
PSP [23]		X	X	X			X	
REMS [24]	X		X	X	X	X	X	
RST [25]		X	X	X		X		Blood glucose
Sepsis Alert [26]		X	X	X			X	Suspected or documented infection, hypoperfusion
SEPSIS [27]	X	X	X	X	X	X	X	Skin appearance
STSS [28]	X		X	X	X	X	X	
Suffoletto [29]		X					X	
UKST red flag* [3]			X	X	X	X	X	Skin appearance

*Excluding lactate, oliguria and recent chemotherapy

National Early Warning Score, version 2 (NEWS2); Quick Sequential Organ Failure Assessment (qSOFA); Critical illness score (CIS); Hamilton Early Warning Score (HEWS); Modified Early Warning Score (MEWS); Prehospital ANTibiotics Against Sepsis (PHANTASi); Paramedic Initiated Treatment of Sepsis Targeting Out-of-hospital Patients clinical trial (PITSTOP); Prehospital Sepsis Assessment Tool (PreSAT); Prehospital Early Sepsis Detection (PRESEP); Prehospital Severe Sepsis (PRESS); Prehospital Sepsis Project (PSP); Rapid Emergency Medicine Score (REMS): Robson Screening Tool (RST); Screening to Enhance Prehospital Identification of Sepsis (SEPSIS); Simple Triage Scoring System (STSS); United Kingdom Sepsis Trust (UKST)

Supplementary Table 2: Agreement between expert doctors during reference standard adjudication

**Assessment**	**Doctor 1**	**Doctor 2**	**Consensus**	**Kappa (95% CI)**
Evidence of infection	86.0%	87.6%	84.7%	0.62 (0.53, 0.71)
SOFA score 2+ worse than normal	60.2%	61.1%	60.0%	0.87 (0.83, 0.91)
Patient meets sepsis-3 criteria	56.0%	55.0%	56.2%	0.89 (0.85, 0.92)
Treatment for sepsis given	52.5%	51.5%	53.3%	0.87 (0.83, 0.91)

Supplementary Table 3: Accuracy of categorised diagnostic impression for the primary reference standard

Threshold	N	TP	FP	FN	TN	Sensitivity	Specificity	PPV	NPV
Sepsis	12870	114	286	234	12236	0.328 (0.28, 0.379)	0.977 (0.974, 0.98)	0.285 (0.243, 0.331)	0.981 (0.979, 0.983)
Sepsis or infection	12870	199	1080	149	11442	0.572 (0.519, 0.623)	0.914 (0.909, 0.919)	0.156 (0.137, 0.176)	0.987 (0.985, 0.989)
Sepsis, infection or nonspecific impression	12870	312	5576	36	6946	0.897 (0.86, 0.924)	0.555 (0.546, 0.563)	0.053 (0.048, 0.059)	0.995 (0.993, 0.996)

Supplementary Table 4: Area under ROC curve for each early warning score for the primary reference standard

Early warning score	Sepsis	Sepsis or infection	Sepsis, infection or nonspecific impression	All diagnostic impressions
NEWS2	0.655 (0.63, 0.68)	0.756 (0.729, 0.783)	0.858 (0.836, 0.88)	0.877 (0.86, 0.895)
qSOFA	0.645 (0.62, 0.669)	0.734 (0.707, 0.761)	0.809 (0.785, 0.834)	0.801 (0.778, 0.824)
90-30-90	0.624 (0.601, 0.648)	0.686 (0.66, 0.712)	0.743 (0.717, 0.769)	0.742 (0.717, 0.768)
Borelli	0.639 (0.615, 0.663)	0.712 (0.686, 0.738)	0.781 (0.755, 0.806)	0.788 (0.764, 0.813)
CIS	0.654 (0.629, 0.679)	0.755 (0.728, 0.782)	0.845 (0.822, 0.867)	0.838 (0.817, 0.859)
HEWS	0.654 (0.629, 0.679)	0.751 (0.724, 0.778)	0.841 (0.818, 0.863)	0.837 (0.816, 0.858)
MEWS	0.654 (0.629, 0.679)	0.753 (0.726, 0.78)	0.851 (0.828, 0.873)	0.857 (0.837, 0.876)
NHS pre-alert	0.624 (0.601, 0.648)	0.696 (0.67, 0.722)	0.751 (0.725, 0.776)	0.747 (0.723, 0.772)
PHANTASi	0.626 (0.602, 0.649)	0.708 (0.682, 0.735)	0.745 (0.719, 0.771)	0.741 (0.716, 0.767)
PITSTOP	0.534 (0.52, 0.547)	0.545 (0.53, 0.56)	0.549 (0.533, 0.564)	0.554 (0.537, 0.57)
PreSAT	0.648 (0.624, 0.673)	0.734 (0.708, 0.761)	0.789 (0.766, 0.813)	0.775 (0.754, 0.797)
PRESEP	0.653 (0.628, 0.678)	0.75 (0.723, 0.777)	0.847 (0.824, 0.87)	0.856 (0.834, 0.878)
PRESS	0.551 (0.534, 0.567)	0.557 (0.54, 0.574)	0.579 (0.559, 0.599)	0.587 (0.566, 0.608)
PSP	0.654 (0.629, 0.679)	0.754 (0.728, 0.781)	0.84 (0.818, 0.862)	0.832 (0.811, 0.852)
REMS	0.654 (0.629, 0.679)	0.752 (0.725, 0.779)	0.814 (0.792, 0.836)	0.757 (0.732, 0.781)
RST	0.648 (0.623, 0.672)	0.737 (0.711, 0.764)	0.782 (0.761, 0.803)	0.73 (0.712, 0.747)
Sepsis alert	0.537 (0.523, 0.551)	0.542 (0.528, 0.557)	0.558 (0.541, 0.576)	0.563 (0.545, 0.581)
SEPSIS	0.654 (0.629, 0.679)	0.755 (0.727, 0.782)	0.862 (0.84, 0.884)	0.882 (0.865, 0.899)
STSS	0.652 (0.627, 0.677)	0.749 (0.722, 0.776)	0.837 (0.814, 0.861)	0.831 (0.809, 0.854)
Suffoletto	0.64 (0.616, 0.664)	0.728 (0.701, 0.754)	0.799 (0.775, 0.823)	0.801 (0.778, 0.824)
UKST red flag	0.648 (0.623, 0.672)	0.733 (0.707, 0.76)	0.788 (0.766, 0.809)	0.756 (0.737, 0.775)

See supplementary table 1 for details of the early warning scores

National Early Warning Score, version 2 (NEWS2); Quick Sequential Organ Failure Assessment (qSOFA); Critical illness score (CIS); Hamilton Early Warning Score (HEWS); Modified Early Warning Score (MEWS); Prehospital ANTibiotics Against Sepsis (PHANTASi); Paramedic Initiated Treatment of Sepsis Targeting Out-of-hospital Patients clinical trial (PITSTOP); Prehospital Sepsis Assessment Tool (PreSAT); Prehospital Early Sepsis Detection (PRESEP); Prehospital Severe Sepsis (PRESS); Prehospital Sepsis Project (PSP); Rapid Emergency Medicine Score (REMS): Robson Screening Tool (RST); Screening to Enhance Prehospital Identification of Sepsis (SEPSIS); Simple Triage Scoring System (STSS); United Kingdom Sepsis Trust (UKST)

Supplementary Table 5: Accuracy of NEWS2 applied only to presentations with a diagnostic impression of sepsis

Threshold	N	TP	FP	FN	TN	Sensitivity	Specificity	PPV	NPV
0	12859	114	282	233	12230	0.329 (0.281, 0.38)	0.977 (0.975, 0.98)	0.288 (0.245, 0.334)	0.981 (0.979, 0.984)
1	12859	114	278	233	12234	0.329 (0.281, 0.38)	0.978 (0.975, 0.98)	0.291 (0.248, 0.338)	0.981 (0.979, 0.984)
2	12859	114	273	233	12239	0.329 (0.281, 0.38)	0.978 (0.975, 0.981)	0.295 (0.251, 0.342)	0.981 (0.979, 0.984)
3	12859	113	262	234	12250	0.326 (0.278, 0.377)	0.979 (0.976, 0.981)	0.301 (0.257, 0.35)	0.981 (0.979, 0.983)
4	12859	111	245	236	12267	0.32 (0.273, 0.371)	0.98 (0.978, 0.983)	0.312 (0.266, 0.362)	0.981 (0.979, 0.983)
5	12859	108	228	239	12284	0.311 (0.265, 0.362)	0.982 (0.979, 0.984)	0.321 (0.274, 0.373)	0.981 (0.978, 0.983)
6	12859	105	190	242	12322	0.303 (0.257, 0.353)	0.985 (0.983, 0.987)	0.356 (0.303, 0.412)	0.981 (0.978, 0.983)
7	12859	95	157	252	12355	0.274 (0.23, 0.323)	0.987 (0.985, 0.989)	0.377 (0.319, 0.438)	0.98 (0.977, 0.982)
8	12859	86	129	261	12383	0.248 (0.205, 0.296)	0.99 (0.988, 0.991)	0.4 (0.337, 0.467)	0.979 (0.977, 0.982)
9	12859	73	94	274	12418	0.21 (0.171, 0.256)	0.992 (0.991, 0.994)	0.437 (0.364, 0.513)	0.978 (0.976, 0.981)
10	12859	59	65	288	12447	0.17 (0.134, 0.213)	0.995 (0.993, 0.996)	0.476 (0.39, 0.563)	0.977 (0.975, 0.98)
11	12859	40	36	307	12476	0.115 (0.086, 0.153)	0.997 (0.996, 0.998)	0.526 (0.416, 0.635)	0.976 (0.973, 0.978)
12	12859	25	21	322	12491	0.072 (0.049, 0.104)	0.998 (0.997, 0.999)	0.543 (0.402, 0.678)	0.975 (0.972, 0.977)
13	12859	8	12	339	12500	0.023 (0.012, 0.045)	0.999 (0.998, 0.999)	0.4 (0.219, 0.613)	0.974 (0.971, 0.976)
14	12859	4	7	343	12505	0.012 (0.004, 0.029)	0.999 (0.999, 1)	0.364 (0.152, 0.646)	0.973 (0.97, 0.976)
15	12859	0	3	347	12509	0 (0, 0.011)	1 (0.999, 1)	0 (0, 0.561)	0.973 (0.97, 0.976)
16	12859	0	0	347	12512	0 (0, 0.011)	1 (1, 1)	-	0.973 (0.97, 0.976)

Supplementary Table 6: Accuracy of qSOFA and other tools applied only to presentations with a diagnostic impression of sepsis

EWS	Threshold	N	TP	FP	FN	TN	Sensitivity	Specificity	PPV	NPV
Pre-alert	0	12870	45	110	303	12412	0.129 (0.098, 0.169)	0.991 (0.989, 0.993)	0.29 (0.225, 0.366)	0.976 (0.973, 0.979)
qSOFA	0	12869	107	249	241	12272	0.307 (0.261, 0.358)	0.98 (0.978, 0.982)	0.301 (0.255, 0.35)	0.981 (0.978, 0.983)
qSOFA	1	12869	72	103	276	12418	0.207 (0.168, 0.253)	0.992 (0.99, 0.993)	0.411 (0.341, 0.485)	0.978 (0.976, 0.981)
qSOFA	2	12869	19	21	329	12500	0.055 (0.035, 0.084)	0.998 (0.997, 0.999)	0.475 (0.329, 0.625)	0.974 (0.971, 0.977)
90-30-90	0	12857	91	169	256	12341	0.262 (0.219, 0.311)	0.986 (0.984, 0.988)	0.35 (0.295, 0.41)	0.98 (0.977, 0.982)
Borelli	0	12835	102	203	245	12285	0.294 (0.248, 0.344)	0.984 (0.981, 0.986)	0.334 (0.284, 0.389)	0.98 (0.978, 0.983)
CIS	0	12855	114	283	233	12225	0.329 (0.281, 0.38)	0.977 (0.975, 0.98)	0.287 (0.245, 0.334)	0.981 (0.979, 0.984)
CIS	4	12855	32	42	315	12466	0.092 (0.066, 0.127)	0.997 (0.995, 0.998)	0.432 (0.326, 0.546)	0.975 (0.973, 0.978)
HEWS	4	12835	101	209	246	12279	0.291 (0.246, 0.341)	0.983 (0.981, 0.985)	0.326 (0.276, 0.38)	0.98 (0.978, 0.983)
MEWS	4	12859	88	189	259	12323	0.254 (0.211, 0.302)	0.985 (0.983, 0.987)	0.318 (0.266, 0.375)	0.979 (0.977, 0.982)
NHS	0	12855	92	204	255	12304	0.265 (0.221, 0.314)	0.984 (0.981, 0.986)	0.311 (0.261, 0.366)	0.98 (0.977, 0.982)
PHANTASi	0	12858	93	210	254	12301	0.268 (0.224, 0.317)	0.983 (0.981, 0.985)	0.307 (0.258, 0.361)	0.98 (0.977, 0.982)
PITSTOP	0	12813	24	20	322	12447	0.069 (0.047, 0.101)	0.998 (0.998, 0.999)	0.545 (0.401, 0.683)	0.975 (0.972, 0.977)
PreSAT	0	12835	110	250	237	12238	0.317 (0.27, 0.368)	0.98 (0.977, 0.982)	0.306 (0.26, 0.355)	0.981 (0.978, 0.983)
PRESEP	3	12835	111	242	236	12246	0.32 (0.273, 0.371)	0.981 (0.978, 0.983)	0.314 (0.268, 0.365)	0.981 (0.979, 0.983)
PRESS	1	12835	35	33	312	12455	0.101 (0.073, 0.137)	0.997 (0.996, 0.998)	0.515 (0.398, 0.629)	0.976 (0.973, 0.978)
PSP	1	12835	111	258	236	12230	0.32 (0.273, 0.371)	0.979 (0.977, 0.982)	0.301 (0.256, 0.349)	0.981 (0.979, 0.983)
REMS	2	12855	114	269	233	12239	0.329 (0.281, 0.38)	0.978 (0.976, 0.981)	0.298 (0.254, 0.345)	0.981 (0.979, 0.984)
RST	0	12857	110	264	237	12246	0.317 (0.27, 0.368)	0.979 (0.976, 0.981)	0.294 (0.25, 0.342)	0.981 (0.978, 0.983)
SAS	0	12836	26	14	321	12475	0.075 (0.052, 0.108)	0.999 (0.998, 0.999)	0.65 (0.495, 0.779)	0.975 (0.972, 0.977)
SEPSIS	4	12856	78	123	269	12386	0.225 (0.184, 0.272)	0.99 (0.988, 0.992)	0.388 (0.323, 0.457)	0.979 (0.976, 0.981)
STSS	1	12855	104	211	243	12297	0.3 (0.254, 0.35)	0.983 (0.981, 0.985)	0.33 (0.281, 0.384)	0.981 (0.978, 0.983)
Suffoletto	0	12813	103	219	243	12248	0.298 (0.252, 0.348)	0.982 (0.98, 0.985)	0.32 (0.271, 0.373)	0.981 (0.978, 0.983)
UK	0	12855	109	237	238	12271	0.314 (0.268, 0.365)	0.981 (0.979, 0.983)	0.315 (0.268, 0.366)	0.981 (0.978, 0.983)

Supplementary Table 7: Accuracy of NEWS2 applied only to presentations with a diagnostic impression of sepsis or infection

Threshold	N	TP	FP	FN	TN	Sensitivity	Specificity	PPV	NPV
0	12859	198	1032	149	11480	0.571 (0.518, 0.622)	0.918 (0.913, 0.922)	0.161 (0.141, 0.183)	0.987 (0.985, 0.989)
1	12859	197	967	150	11545	0.568 (0.515, 0.619)	0.923 (0.918, 0.927)	0.169 (0.149, 0.192)	0.987 (0.985, 0.989)
2	12859	197	889	150	11623	0.568 (0.515, 0.619)	0.929 (0.924, 0.933)	0.181 (0.16, 0.205)	0.987 (0.985, 0.989)
3	12859	191	776	156	11736	0.55 (0.498, 0.602)	0.938 (0.934, 0.942)	0.198 (0.174, 0.224)	0.987 (0.985, 0.989)
4	12859	181	658	166	11854	0.522 (0.469, 0.574)	0.947 (0.943, 0.951)	0.216 (0.189, 0.245)	0.986 (0.984, 0.988)
5	12859	171	540	176	11972	0.493 (0.441, 0.545)	0.957 (0.953, 0.96)	0.241 (0.211, 0.273)	0.986 (0.983, 0.987)
6	12859	155	410	192	12102	0.447 (0.395, 0.499)	0.967 (0.964, 0.97)	0.274 (0.239, 0.313)	0.984 (0.982, 0.986)
7	12859	133	314	214	12198	0.383 (0.334, 0.435)	0.975 (0.972, 0.978)	0.298 (0.257, 0.342)	0.983 (0.98, 0.985)
8	12859	109	218	238	12294	0.314 (0.268, 0.365)	0.983 (0.98, 0.985)	0.333 (0.284, 0.386)	0.981 (0.978, 0.983)
9	12859	91	146	256	12366	0.262 (0.219, 0.311)	0.988 (0.986, 0.99)	0.384 (0.324, 0.447)	0.98 (0.977, 0.982)
10	12859	68	91	279	12421	0.196 (0.158, 0.241)	0.993 (0.991, 0.994)	0.428 (0.353, 0.505)	0.978 (0.975, 0.98)
11	12859	47	48	300	12464	0.135 (0.103, 0.175)	0.996 (0.995, 0.997)	0.495 (0.396, 0.594)	0.976 (0.974, 0.979)
12	12859	29	28	318	12484	0.084 (0.059, 0.117)	0.998 (0.997, 0.998)	0.509 (0.383, 0.634)	0.975 (0.972, 0.978)
13	12859	11	17	336	12495	0.032 (0.018, 0.056)	0.999 (0.998, 0.999)	0.393 (0.236, 0.576)	0.974 (0.971, 0.976)
14	12859	5	8	342	12504	0.014 (0.006, 0.033)	0.999 (0.999, 1)	0.385 (0.177, 0.645)	0.973 (0.97, 0.976)
15	12859	0	3	347	12509	0 (0, 0.011)	1 (0.999, 1)	0 (0, 0.561)	0.973 (0.97, 0.976)
16	12859	0	0	347	12512	0 (0, 0.011)	1 (1, 1)	-	0.973 (0.97, 0.976)

Supplementary Table 8: Accuracy of qSOFA and other tools applied only to presentations with a diagnostic impression of sepsis or infection

EWS	Threshold	N	TP	FP	FN	TN	Sensitivity	Specificity	PPV	NPV
Pre-alert	0	12870	52	133	296	12389	0.149 (0.116, 0.191)	0.989 (0.987, 0.991)	0.281 (0.221, 0.35)	0.977 (0.974, 0.979)
qSOFA	0	12869	180	758	168	11763	0.517 (0.465, 0.569)	0.939 (0.935, 0.944)	0.192 (0.168, 0.218)	0.986 (0.984, 0.988)
qSOFA	1	12869	106	192	242	12329	0.305 (0.259, 0.355)	0.985 (0.982, 0.987)	0.356 (0.304, 0.412)	0.981 (0.978, 0.983)
qSOFA	2	12869	23	22	325	12499	0.066 (0.044, 0.097)	0.998 (0.997, 0.999)	0.511 (0.37, 0.65)	0.975 (0.972, 0.977)
90-30-90	0	12857	140	387	207	12123	0.403 (0.353, 0.456)	0.969 (0.966, 0.972)	0.266 (0.23, 0.305)	0.983 (0.981, 0.985)
Borelli	0	12835	160	463	187	12025	0.461 (0.409, 0.514)	0.963 (0.959, 0.966)	0.257 (0.224, 0.293)	0.985 (0.982, 0.987)
CIS	0	12855	198	1039	149	11469	0.571 (0.518, 0.622)	0.917 (0.912, 0.922)	0.16 (0.141, 0.182)	0.987 (0.985, 0.989)
CIS	4	12855	38	54	309	12454	0.11 (0.081, 0.147)	0.996 (0.994, 0.997)	0.413 (0.318, 0.515)	0.976 (0.973, 0.978)
HEWS	4	12835	154	477	193	12011	0.444 (0.392, 0.496)	0.962 (0.958, 0.965)	0.244 (0.212, 0.279)	0.984 (0.982, 0.986)
MEWS	4	12859	135	412	212	12100	0.389 (0.339, 0.441)	0.967 (0.964, 0.97)	0.247 (0.213, 0.285)	0.983 (0.98, 0.985)
NHS	0	12855	149	471	198	12037	0.429 (0.378, 0.482)	0.962 (0.959, 0.966)	0.24 (0.208, 0.275)	0.984 (0.981, 0.986)
PHANTASi	0	12858	161	588	186	11923	0.464 (0.412, 0.517)	0.953 (0.949, 0.957)	0.215 (0.187, 0.246)	0.985 (0.982, 0.987)
PITSTOP	0	12813	32	28	314	12439	0.092 (0.066, 0.128)	0.998 (0.997, 0.998)	0.533 (0.409, 0.654)	0.975 (0.973, 0.978)
PreSAT	0	12835	183	732	164	11756	0.527 (0.475, 0.579)	0.941 (0.937, 0.945)	0.2 (0.175, 0.227)	0.986 (0.984, 0.988)
PRESEP	3	12835	183	738	164	11750	0.527 (0.475, 0.579)	0.941 (0.937, 0.945)	0.199 (0.174, 0.226)	0.986 (0.984, 0.988)
PRESS	1	12835	40	61	307	12427	0.115 (0.086, 0.153)	0.995 (0.994, 0.996)	0.396 (0.306, 0.494)	0.976 (0.973, 0.978)
PSP	1	12835	188	723	159	11765	0.542 (0.489, 0.593)	0.942 (0.938, 0.946)	0.206 (0.181, 0.234)	0.987 (0.984, 0.989)
REMS	2	12855	197	987	150	11521	0.568 (0.515, 0.619)	0.921 (0.916, 0.926)	0.166 (0.146, 0.189)	0.987 (0.985, 0.989)
RST	0	12857	188	836	159	11674	0.542 (0.489, 0.593)	0.933 (0.929, 0.937)	0.184 (0.161, 0.208)	0.987 (0.984, 0.988)
SAS	0	12836	30	22	317	12467	0.086 (0.061, 0.121)	0.998 (0.997, 0.999)	0.577 (0.442, 0.701)	0.975 (0.972, 0.978)
SEPSIS	4	12856	107	216	240	12293	0.308 (0.262, 0.359)	0.983 (0.98, 0.985)	0.331 (0.282, 0.384)	0.981 (0.978, 0.983)
STSS	1	12855	174	607	173	11901	0.501 (0.449, 0.554)	0.951 (0.948, 0.955)	0.223 (0.195, 0.253)	0.986 (0.983, 0.988)
Suffoletto	0	12813	176	669	170	11798	0.509 (0.456, 0.561)	0.946 (0.942, 0.95)	0.208 (0.182, 0.237)	0.986 (0.984, 0.988)
UK	0	12855	181	686	166	11822	0.522 (0.469, 0.574)	0.945 (0.941, 0.949)	0.209 (0.183, 0.237)	0.986 (0.984, 0.988)

Supplementary Table 9: Accuracy of NEWS2 applied only to presentations with a diagnostic impression of sepsis, infection, or nonspecific presentation

Threshold	N	TP	FP	FN	TN	Sensitivity	Specificity	PPV	NPV
0	12859	307	4638	40	7874	0.885 (0.847, 0.914)	0.629 (0.621, 0.638)	0.062 (0.056, 0.069)	0.995 (0.993, 0.996)
1	12859	302	3833	45	8679	0.87 (0.831, 0.902)	0.694 (0.686, 0.702)	0.073 (0.065, 0.081)	0.995 (0.993, 0.996)
2	12859	297	3248	50	9264	0.856 (0.815, 0.889)	0.74 (0.733, 0.748)	0.084 (0.075, 0.093)	0.995 (0.993, 0.996)
3	12859	287	2598	60	9914	0.827 (0.784, 0.863)	0.792 (0.785, 0.799)	0.099 (0.089, 0.111)	0.994 (0.992, 0.995)
4	12859	270	2048	77	10464	0.778 (0.731, 0.819)	0.836 (0.83, 0.843)	0.116 (0.104, 0.13)	0.993 (0.991, 0.994)
5	12859	252	1612	95	10900	0.726 (0.677, 0.77)	0.871 (0.865, 0.877)	0.135 (0.12, 0.151)	0.991 (0.989, 0.993)
6	12859	220	1152	127	11360	0.634 (0.582, 0.683)	0.908 (0.903, 0.913)	0.16 (0.142, 0.181)	0.989 (0.987, 0.991)
7	12859	181	823	166	11689	0.522 (0.469, 0.574)	0.934 (0.93, 0.938)	0.18 (0.158, 0.205)	0.986 (0.984, 0.988)
8	12859	147	513	200	11999	0.424 (0.373, 0.476)	0.959 (0.955, 0.962)	0.223 (0.193, 0.256)	0.984 (0.981, 0.986)
9	12859	111	311	236	12201	0.32 (0.273, 0.371)	0.975 (0.972, 0.978)	0.263 (0.223, 0.307)	0.981 (0.978, 0.983)
10	12859	82	181	265	12331	0.236 (0.195, 0.284)	0.986 (0.983, 0.987)	0.312 (0.259, 0.37)	0.979 (0.976, 0.981)
11	12859	55	90	292	12422	0.159 (0.124, 0.201)	0.993 (0.991, 0.994)	0.379 (0.304, 0.46)	0.977 (0.974, 0.979)
12	12859	34	47	313	12465	0.098 (0.071, 0.134)	0.996 (0.995, 0.997)	0.42 (0.318, 0.528)	0.976 (0.973, 0.978)
13	12859	12	28	335	12484	0.035 (0.02, 0.059)	0.998 (0.997, 0.998)	0.3 (0.181, 0.454)	0.974 (0.971, 0.976)
14	12859	6	12	341	12500	0.017 (0.008, 0.037)	0.999 (0.998, 0.999)	0.333 (0.163, 0.563)	0.973 (0.971, 0.976)
15	12859	0	3	347	12509	0 (0, 0.011)	1 (0.999, 1)	0 (0, 0.561)	0.973 (0.97, 0.976)
16	12859	0	0	347	12512	0 (0, 0.011)	1 (1, 1)	-	0.973 (0.97, 0.976)

Supplementary Table 10: Accuracy of qSOFA and other tools applied only to presentations with a diagnostic impression of sepsis, infection, or nonspecific presentation

EWS	Threshold	N	TP	FP	FN	TN	Sensitivity	Specificity	PPV	NPV
Pre-alert	0	12870	71	313	277	12209	0.204 (0.165, 0.249)	0.975 (0.972, 0.978)	0.185 (0.149, 0.227)	0.978 (0.975, 0.98)
qSOFA	0	12869	274	2984	74	9537	0.787 (0.741, 0.827)	0.762 (0.754, 0.769)	0.084 (0.075, 0.094)	0.992 (0.99, 0.994)
qSOFA	1	12869	149	551	199	11970	0.428 (0.377, 0.481)	0.956 (0.952, 0.959)	0.213 (0.184, 0.245)	0.984 (0.981, 0.986)
qSOFA	2	12869	32	45	316	12476	0.092 (0.066, 0.127)	0.996 (0.995, 0.997)	0.416 (0.312, 0.527)	0.975 (0.972, 0.978)
90-30-90	0	12857	209	1463	138	11047	0.602 (0.55, 0.652)	0.883 (0.877, 0.889)	0.125 (0.11, 0.142)	0.988 (0.985, 0.99)
Borelli	0	12835	227	1160	120	11328	0.654 (0.603, 0.702)	0.907 (0.902, 0.912)	0.164 (0.145, 0.184)	0.99 (0.987, 0.991)
CIS	0	12855	310	5150	37	7358	0.893 (0.856, 0.922)	0.588 (0.58, 0.597)	0.057 (0.051, 0.063)	0.995 (0.993, 0.996)
CIS	4	12855	51	136	296	12372	0.147 (0.114, 0.188)	0.989 (0.987, 0.991)	0.273 (0.214, 0.341)	0.977 (0.974, 0.979)
HEWS	4	12835	219	1381	128	11107	0.631 (0.579, 0.68)	0.889 (0.884, 0.895)	0.137 (0.121, 0.155)	0.989 (0.986, 0.99)
MEWS	4	12859	177	909	170	11603	0.51 (0.458, 0.562)	0.927 (0.923, 0.932)	0.163 (0.142, 0.186)	0.986 (0.983, 0.988)
NHS	0	12855	221	1696	126	10812	0.637 (0.585, 0.686)	0.864 (0.858, 0.87)	0.115 (0.102, 0.13)	0.988 (0.986, 0.99)
PHANTASi	0	12858	204	1221	143	11290	0.588 (0.535, 0.638)	0.902 (0.897, 0.907)	0.143 (0.126, 0.162)	0.987 (0.985, 0.989)
PITSTOP	0	12813	35	51	311	12416	0.101 (0.074, 0.137)	0.996 (0.995, 0.997)	0.407 (0.309, 0.513)	0.976 (0.973, 0.978)
PreSAT	0	12835	258	2060	89	10428	0.744 (0.695, 0.787)	0.835 (0.828, 0.841)	0.111 (0.099, 0.125)	0.992 (0.99, 0.993)
PRESEP	3	12835	255	1736	92	10752	0.735 (0.686, 0.779)	0.861 (0.855, 0.867)	0.128 (0.114, 0.143)	0.992 (0.99, 0.993)
PRESS	1	12835	58	163	289	12325	0.167 (0.132, 0.21)	0.987 (0.985, 0.989)	0.262 (0.209, 0.324)	0.977 (0.974, 0.98)
PSP	1	12835	268	2429	79	10059	0.772 (0.725, 0.813)	0.805 (0.798, 0.812)	0.099 (0.089, 0.111)	0.992 (0.99, 0.994)
REMS	2	12855	309	4919	38	7589	0.89 (0.853, 0.919)	0.607 (0.598, 0.615)	0.059 (0.053, 0.066)	0.995 (0.993, 0.996)
RST	0	12857	279	3008	68	9502	0.804 (0.759, 0.842)	0.76 (0.752, 0.767)	0.085 (0.076, 0.095)	0.993 (0.991, 0.994)
SAS	0	12836	42	52	305	12437	0.121 (0.091, 0.16)	0.996 (0.995, 0.997)	0.447 (0.35, 0.547)	0.976 (0.973, 0.979)
SEPSIS	4	12856	137	427	210	12082	0.395 (0.345, 0.447)	0.966 (0.963, 0.969)	0.243 (0.209, 0.28)	0.983 (0.98, 0.985)
STSS	1	12855	262	2226	85	10282	0.755 (0.707, 0.797)	0.822 (0.815, 0.829)	0.105 (0.094, 0.118)	0.992 (0.99, 0.993)
Suffoletto	0	12813	247	1451	99	11016	0.714 (0.664, 0.759)	0.884 (0.878, 0.889)	0.145 (0.129, 0.163)	0.991 (0.989, 0.993)
UK	0	12855	275	2720	72	9788	0.793 (0.747, 0.832)	0.783 (0.775, 0.79)	0.092 (0.082, 0.103)	0.993 (0.991, 0.994)

Supplementary Table 11: Accuracy of NEWS2 applied to all presentations

Threshold	N	TP	FP	FN	TN	Sensitivity	Specificity	PPV	NPV
0	12859	342	9189	5	3323	0.986 (0.967, 0.994)	0.266 (0.258, 0.273)	0.036 (0.032, 0.04)	0.998 (0.996, 0.999)
1	12859	332	6749	15	5763	0.957 (0.93, 0.974)	0.461 (0.452, 0.469)	0.047 (0.042, 0.052)	0.997 (0.996, 0.998)
2	12859	326	5210	21	7302	0.939 (0.909, 0.96)	0.584 (0.575, 0.592)	0.059 (0.053, 0.065)	0.997 (0.996, 0.998)
3	12859	312	3801	35	8711	0.899 (0.863, 0.927)	0.696 (0.688, 0.704)	0.076 (0.068, 0.084)	0.996 (0.994, 0.997)
4	12859	290	2792	57	9720	0.836 (0.793, 0.871)	0.777 (0.769, 0.784)	0.094 (0.084, 0.105)	0.994 (0.992, 0.995)
5	12859	271	2088	76	10424	0.781 (0.735, 0.821)	0.833 (0.826, 0.84)	0.115 (0.103, 0.128)	0.993 (0.991, 0.994)
6	12859	235	1460	112	11052	0.677 (0.626, 0.724)	0.883 (0.878, 0.889)	0.139 (0.123, 0.156)	0.99 (0.988, 0.992)
7	12859	196	1018	151	11494	0.565 (0.512, 0.616)	0.919 (0.914, 0.923)	0.161 (0.142, 0.183)	0.987 (0.985, 0.989)
8	12859	155	616	192	11896	0.447 (0.395, 0.499)	0.951 (0.947, 0.954)	0.201 (0.174, 0.231)	0.984 (0.982, 0.986)
9	12859	117	364	230	12148	0.337 (0.289, 0.388)	0.971 (0.968, 0.974)	0.243 (0.207, 0.284)	0.981 (0.979, 0.984)
10	12859	86	214	261	12298	0.248 (0.205, 0.296)	0.983 (0.98, 0.985)	0.287 (0.238, 0.34)	0.979 (0.977, 0.982)
11	12859	57	110	290	12402	0.164 (0.129, 0.207)	0.991 (0.989, 0.993)	0.341 (0.274, 0.416)	0.977 (0.974, 0.98)
12	12859	36	57	311	12455	0.104 (0.076, 0.14)	0.995 (0.994, 0.996)	0.387 (0.294, 0.489)	0.976 (0.973, 0.978)
13	12859	13	35	334	12477	0.037 (0.022, 0.063)	0.997 (0.996, 0.998)	0.271 (0.166, 0.41)	0.974 (0.971, 0.977)
14	12859	6	15	341	12497	0.017 (0.008, 0.037)	0.999 (0.998, 0.999)	0.286 (0.138, 0.5)	0.973 (0.971, 0.976)
15	12859	0	4	347	12508	0 (0, 0.011)	1 (0.999, 1)	0 (0, 0.49)	0.973 (0.97, 0.976)
16	12859	0	1	347	12511	0 (0, 0.011)	1 (1, 1)	0 (0, 0.793)	0.973 (0.97, 0.976)

Supplementary Table 12: Accuracy of qSOFA and other tools applied to all presentations

EWS	Threshold	N	TP	FP	FN	TN	Sensitivity	Specificity	PPV	NPV
Pre-alert	0	12870	80	531	268	11991	0.23 (0.189, 0.277)	0.958 (0.954, 0.961)	0.131 (0.106, 0.16)	0.978 (0.975, 0.981)
qSOFA	0	12869	301	4908	47	7613	0.865 (0.825, 0.897)	0.608 (0.599, 0.617)	0.058 (0.052, 0.064)	0.994 (0.992, 0.995)
qSOFA	1	12869	160	790	188	11731	0.46 (0.408, 0.512)	0.937 (0.933, 0.941)	0.168 (0.146, 0.194)	0.984 (0.982, 0.986)
qSOFA	2	12869	33	65	315	12456	0.095 (0.068, 0.13)	0.995 (0.993, 0.996)	0.337 (0.251, 0.435)	0.975 (0.972, 0.978)
90-30-90	0	12857	222	1937	125	10573	0.64 (0.588, 0.688)	0.845 (0.839, 0.851)	0.103 (0.091, 0.116)	0.988 (0.986, 0.99)
Borelli	0	12835	242	1505	105	10983	0.697 (0.647, 0.743)	0.879 (0.874, 0.885)	0.139 (0.123, 0.156)	0.991 (0.989, 0.992)
CIS	0	12855	344	10864	3	1644	0.991 (0.975, 0.997)	0.131 (0.126, 0.137)	0.031 (0.028, 0.034)	0.998 (0.995, 0.999)
CIS	4	12855	57	171	290	12337	0.164 (0.129, 0.207)	0.986 (0.984, 0.988)	0.25 (0.198, 0.31)	0.977 (0.974, 0.98)
HEWS	4	12835	235	1923	112	10565	0.677 (0.626, 0.724)	0.846 (0.84, 0.852)	0.109 (0.096, 0.123)	0.99 (0.987, 0.991)
MEWS	4	12859	190	1232	157	11280	0.548 (0.495, 0.599)	0.902 (0.896, 0.907)	0.134 (0.117, 0.152)	0.986 (0.984, 0.988)
NHS	0	12855	241	2500	106	10008	0.695 (0.644, 0.741)	0.8 (0.793, 0.807)	0.088 (0.078, 0.099)	0.99 (0.987, 0.991)
PHANTASi	0	12858	215	1710	132	10801	0.62 (0.567, 0.669)	0.863 (0.857, 0.869)	0.112 (0.098, 0.127)	0.988 (0.986, 0.99)
PITSTOP	0	12813	39	67	307	12400	0.113 (0.084, 0.15)	0.995 (0.993, 0.996)	0.368 (0.282, 0.463)	0.976 (0.973, 0.978)
PreSAT	0	12835	277	3099	70	9389	0.798 (0.753, 0.837)	0.752 (0.744, 0.759)	0.082 (0.073, 0.092)	0.993 (0.991, 0.994)
PRESEP	3	12835	270	2272	77	10216	0.778 (0.731, 0.819)	0.818 (0.811, 0.825)	0.106 (0.095, 0.119)	0.993 (0.991, 0.994)
PRESS	1	12835	65	212	282	12276	0.187 (0.15, 0.232)	0.983 (0.981, 0.985)	0.235 (0.189, 0.288)	0.978 (0.975, 0.98)
PSP	1	12835	292	4418	55	8070	0.841 (0.799, 0.876)	0.646 (0.638, 0.655)	0.062 (0.055, 0.069)	0.993 (0.991, 0.995)
REMS	2	12855	343	10288	4	2220	0.988 (0.971, 0.996)	0.177 (0.171, 0.184)	0.032 (0.029, 0.036)	0.998 (0.995, 0.999)
RST	0	12857	304	5217	43	7293	0.876 (0.837, 0.907)	0.583 (0.574, 0.592)	0.055 (0.049, 0.061)	0.994 (0.992, 0.996)
SAS	0	12836	46	78	301	12411	0.133 (0.101, 0.172)	0.994 (0.992, 0.995)	0.371 (0.291, 0.459)	0.976 (0.974, 0.979)
SEPSIS	4	12856	143	490	204	12019	0.412 (0.362, 0.465)	0.961 (0.957, 0.964)	0.226 (0.195, 0.26)	0.983 (0.981, 0.985)
STSS	1	12855	282	3326	65	9182	0.813 (0.768, 0.85)	0.734 (0.726, 0.742)	0.078 (0.07, 0.087)	0.993 (0.991, 0.994)
Suffoletto	0	12813	263	1969	83	10498	0.76 (0.712, 0.802)	0.842 (0.836, 0.848)	0.118 (0.105, 0.132)	0.992 (0.99, 0.994)
UK	0	12855	297	4291	50	8217	0.856 (0.815, 0.889)	0.657 (0.649, 0.665)	0.065 (0.058, 0.072)	0.994 (0.992, 0.995)
