# Supplementary material for: Prehospital early warning scores for adults with suspected sepsis: retrospective diagnostic cohort study
Source: Emerg Med J. 2023 Sep 6;40(11):768–76. doi: 10.1136/emermed-2023-213315 (PMC10646863; doi:10.1136/emermed-2023-213315)
Supplement: Supplementary data [file emermed-2023-213315supp001.pdf]

Supplementary Appendix 1: Categorisation of diagnostic impressions

Category	Diagnostic impression
1	Sepsis
2	Cold & flu, Febrile illness, Meningitis, Pyrexia of unknown origin, Chest infection-pneumonia, UTI
3	Catheter problems, COPD, Convulsion/Fitting, Collapse-reason unknown, Confused/distressed/upset, Diarrhoea/Constipation, Dizzy/near faint/ loss of coordination, Shortness of Breath, Generally unwell, Haematuria, Headache, Hypotension, Other medical condition, Urinary Retention, Shock (hypovolemic), Transient Loss of Consciousness, Unconscious, Vomiting
4	Abdominal pain, Alcohol related, Allergic reaction/rash, Anaphylactic shock, AAA, Asthma, Bite/sting, Bleeding PR, Bleeding PV, Cardiac arrest, Cardiac STEMI, Cardiac chest pain (ACS), Cardiac NSTEMI, Choking, Carbon monoxide poisoning, Dental, Drug overdose, End of life care / Palliative, Epileptic fit, Epistaxis, Eye injury/eye problem, Falls, Gynaecological, Haematemesis, Haemoptysis, Haemorrhage/lacerations, Hyperglycaemia, Hypertension, Hypoglycaemia, Neurological problems, Pain – back non-traumatic, Pain – other, Panic attack, Poisoning, Pulmonary embolism, Rape/sexual assault, Pneumothorax (spontaneous), Renal problems/colic, Respiratory arrest, Seizures (non-EP), Smoke inhalation, Solvent related, Stroke – FAST positive, Unable to cope, No injury or illness, Cardiac Arrhythmia, Vascular Emergency (Non AAA), Dead on EMS arrival - signs inconsistent with life, Resuscitation unsuccessful
Excluded	Burns, Drowning, Electrocution, Fracture/possible fracture, Hanging, Head injury, Major trauma, Minor cuts & bruising, Minor injuries – other, Multiple injuries<br>Obstetric – BBA, Obstetric – birth imminent, Obstetric – miscarriage, Obstetric – normal labour, Obstetric – premature labour, Obstetric emergency (other), Psychiatric problems, Spinal injury, Sprain/strain/dislocation, Stabbed/shot/weapon wound, Wound Closure, Non accidental injury

Supplementary Appendix 2: Details of each early warning score

90-30-90

Dichotomous assessment, positive if any of the following criteria are met:

1. Systolic BP < 90mmHg
2. Respiratory rate > 30/minute
3. Oxygen saturation < 90%

Modification:
If the oxygen saturation is measured on supplemental oxygen, it is assumed to be < 90% on air (i.e. the criteria is positive).*

Missing data:
Assume any missing criterion is negative/normal.

The Borelli strategy

Dichotomous assessment, positive if three or more criteria are met:

- Respiratory rate > 20/minute
- Heart rate > 90/minute
- Systolic BP < 90 mmHg
- Documented fever or temperature >38.3°C or <36°C
- New onset of mental status change
- O2 saturation < 90%
- Suspected infection

Modification:
Documented fever or temperature >38.3°C or <36°C, is effectively just temperature >38.3°C or <36°C
New onset of mental status change assumed if the GCS verbal scale is <5. If the GCS verbal scale is missing, then mental status change is assumed if GCS<15 or AVPU<A.
If the oxygen saturation is measured on supplemental oxygen, it is assumed to be < 90% on air (i.e. the criteria is positive).

Missing data:
Assume any missing variable is negative

Critical illness score (CIS)

Score	0	1	2
Respiratory Rate	12-23	<12 or 24-35	>35
Heart Rate	<120	≥120	
Systolic BP	>90	≤90	
Age	<45	≥45	
SpO2	≥88	<88	
GCS	15	8-14	<8

Thresholds of >4 or >0 are suggested, depending upon whether specificity or sensitivity are to be optimised

Modification:
If the oxygen saturation is measured on supplemental oxygen, it is assumed to be < 88% on air (i.e. scores 1 point).

Missing data:
Assume any missing variable scores zero.

Hamilton Early Warning Score (HEWS)

Score	3	2	1	0	1	2	3
Respiratory Rate	<8	8-13		14-20		21-30	>30
Oxygen saturation	<85		85-91	>91			
Heart Rate		<40	40-50	51-100	101-110	111-130	>130
Systolic BP	<70	71-90		91-170		171-200	>200
Temperature	<35.0		35.1-36.4	36.5-38.0	38.1-39.0	>39.0	
Neurology				Alert	Voice	Pain	Unresponsive
Air or Oxygen				Air	≤ 5 L/min or ≤ 50% by mask		>5 L/min or >50% by mask

*CAM positive removed as not routinely recorded

Threshold > 4

Modification:
If AVPU is missing, infer from GCS.
If on oxygen but amount unknown, score 2 points

Missing data:
Assume any missing variable scores zero

Modified Early Warning Score (MEWS)

MEWS has five parameters, each of which are scored from zero to two or three providing an overall score between zero and 14.

Score	3	2	1	0	1	2	3
Respiratory Rate		<9		9-14	15-20	21-29	≥30
Heart Rate		≤40	41-50	51-100	101-110	111-129	≥130
Systolic BP	≤70	71-80	81-100	101-199		≥200	
Temperature		<35.0		35.0-38.4		≥38.5	
AVPU				Alert	Voice	Pain	Unresponsive

A threshold of 5 or more has been shown to be associated with an increased risk of death

Modification:
If AVPU missing, infer AVPU from GCS

Missing data:
Assume any missing variable scores zero.

National Early Warning Score, version 2 (NEWS2)

The NEWS2 has seven parameters, each of which are scored from zero to three providing an overall score between zero and 20.

Score	3	2	1	0	1	2	3
Respiratory Rate	≤8		9-11	12-20		21-24	≥25
Oxygen saturation	≤91	92-93	94-95	≥96			
Heart Rate	≤40		41-50	51-90	91-110	111-130	≥131
Systolic BP	≤90	91-100	101-110	111-219			≥220
Temperature	≤35.0		35.1-36.0	36.1-38.0	38.1-39.0	≥39.1	
Neurology				Alert			Confusion, Voice, Pain, Unresponsive
Air or Oxygen		Oxygen (based on FiO_2>21%, or FiO_2>0 L/min)		Air			

We will not use the scale for patients with confirmed hypercapnic respiratory failure.

Modification:
If AVPU is missing, infer AVPU from GCS

Missing data:
Assume any missing variable scores zero.

NHS pre-alert

Pre-alert if any of the following are present:

- Respiratory rate ≤8 or ≥25
- O2 saturations on oxygen <92% (Patients usually running normal oxygen saturations) <84% (Patients with chronic hypercapnic respiratory failure)
- Systolic <90mmHg OR downward-trending systolic where symptomatic
- Tachycardia ≥131
- GCS motor <4

Sepsis red flag criteria evaluated as part of UK Sepsis Trust criteria

Modification:
Drop <84% oxygen saturation threshold for patients with chronic hypercapnic respiratory failure
Drop downward-trending systolic where symptomatic

Missing data:
Assume any missing criterion is negative

Prehospital ANTibiotics Against Sepsis (PHANTASi)

Dichotomous assessment, positive if both the following criteria are met:

1. Temperature >38 °C or < 36 °C
2. Heart rate > 90 beats per minute or respiratory rate > 20 per minutes

Modification:
None required

Missing data:
Assume any missing variable is negative/normal.

Paramedic Initiated Treatment of Sepsis Targeting Out-of-hospital Patients clinical trial (PITSTOP)

Dichotomous assessment, positive if all the following three criteria are met:

1. Paramedic suspects possible infection
2. Temperature ≥ 38.0 °C
3. Systolic BP < 100mmHg

Modification:
None required

Missing data:
Assume any missing criterion is negative/normal.

Prehospital Sepsis Assessment Tool (PreSAT)

Dichotomous assessment, positive if both the following criteria are met:

1. Presentation suggestive of infection
2. Any two from (a) temperature >38°C or <36°C, (b) heart rate > 90/min, (c) respiratory rate > 20/min, (d) systolic BP < 90mmHg

Modification:
None required

Missing data:
Assume any missing variable is negative/normal.

Prehospital Early Sepsis Detection (PRESEP)

Parameter	Score
Temperature > 38°C	4
Temperature < 36°C	1
SaO2 < 92%	2
RR > 22 breaths/min	1
HR > 90 beats/min	2
BP < 90 mm Hg	2

Recommended threshold >3

Modification:
If the oxygen saturation is measured on supplemental oxygen, it is assumed to be < 92% on air (i.e. score 2 points).

Missing data:
Assume any missing variable scores zero.

Prehospital Severe Sepsis (PRESS)

The score is only applied to patients meeting all three of the following criteria, so patients not meeting these criteria should score zero:

- Heart rate >90/min
- Respiratory rate >20/min
- Systolic blood pressure BP <110 mmHg

Score	0	1	2	3	4	5
Age	<40		≥60		40-59	
SpO2	≥90	80-89		70-79	60-69	<60
Systolic BP	100-109	90-99	80-89	70-79	60-69	<60
Hot tactile temperature				X		
ED chief concern: sick person				X		
Nursing home transport					X	

Threshold > 1

Modification:
If the oxygen saturation is measured on supplemental oxygen, it is assumed to be < 90% on air and scored 2 points.
Infer hot tactile temperature from recorded temperature > 38°C
Drop ED chief concern sick person – address this through diagnostic impression
Drop nursing home transport

Missing data:
Assume any missing variable scores zero

Prehospital Sepsis Project (PSP)

Parameter	Score
Temperature > 38°C	1
Heart rate / systolic BP ≥ 0.7	2
Respiratory rate > 22/min	1

Low risk = 0-1 point, moderate risk = 2 points, high risk = 3-4 points

Modification:
None required

Missing data:
Assume missing temperature or respiratory rate scores zero.
Assume heart rate / systolic BP scores zero unless either (a) heart rate is > 100 and systolic BP is missing, or (b) systolic BP is <100 and heart rate is missing.

qSOFA

Parameter	Score
GCS <15	1
Respiratory rate ≥22	1
Systolic BP ≤100	1

Total score 0-3
Low risk = 0 or 1
High risk = 2 or 3

Modification:
None required

Missing data:
Assume any missing variable scores zero

Rapid Emergency Medicine Score (REMS)

Score	0	1	2	3	4	5	6
Age	<45		45-54	55-64		65-74	>65
MAP	70-109		50-69 or 110-129	130-159	>159 or <50		
Heart rate	70-109		55-69 or 110-139	40-54 or 140-179	<179 or <40		
Respiratory rate	12-24	10-11 or 25-34	6-9	35-49	<6 or >49		
SpO2	>89%	86-89%		75-85%	<75%		
GCS	>13	11-13	8-10	5-7	3-4		

High risk (REMS ≥3): patient may need aggressive treatment
Low risk (REMS <3): patient may be appropriate to triage for routine treatment

Modification:
If the oxygen saturation is measured on supplemental oxygen, it is assumed to be < 89% on air and scored 2 points.

Missing data:
Assume any missing variable scores zero

Robson Screening Tool (RST)

Dichotomous assessment, positive if presentation suggestive of infection and any two of:

1. Temperature > 38.3° or < 36°
2. Heart rate > 90 beats/min
3. Respiratory rate > 20 breaths/min
4. Acutely altered mental status
5. Plasma glucose > 6.6 mmol/l (unless diabetic)

Modification:
Criterion 5 applies regardless of whether they are diabetic
Acutely altered mental status change assumed if the GCS verbal scale is <5. If the GCS verbal scale is missing, then acutely altered mental status is assumed if GCS<15 or AVPU=A.

Missing data:
Assume any missing criterion is negative

Screening to Enhance Prehospital Identification of Sepsis (SEPSIS)

Parameter	-1	0	1	2
Age		≤60	>60	
Respiratory rate		≤20 or >60	21-40	40-60
SpO2		≥94	<94	
Heart rate		≤100	101-140	141-160
Systolic BP	>160	<60 or 100-160	60-99	
GCS		13-15	3-12	
Temperature		<37.5	37.5 to 39.5	>39.5
Skin			Jaundice, pallor, mottling	

High risk if score > 4

Modification:
Skin features dropped from the score (not recorded on ePFR)

Missing data:
Assume any missing variable scores zero
Give 1 point if SpO2>94% on oxygen

Sepsis Alert

At least two systematic inflammatory response syndrome (SIRS) criteria:

- Temperature > 38C or < 36C
- Pulse > 90 beats/min
- Respiratory rate > 20 breaths/min or mechanically ventilated

And

Suspected or documented infection

And

Hypoperfusion, as manifested by one of the following:

- Systolic BP < 90 mm Hg
- Mean arterial pressure < 65 mm Hg
- Lactate level ≥ 4 mmol/L

Modification:
Drop lactate level ≥ 4 mmol/L

Missing data:
Assume negative if missing

Simple Triage Scoring System (STSS)

Parameter	Score
Age >65 yrs	1
Altered mental status	1
Respiratory rate of >30 breaths/min	1
Low oxygen saturation	1
Shock index of >1 (heart rate > systolic BP)	1

Modification:
Altered mental status change assumed if the GCS verbal scale is <5. If the GCS verbal scale is missing, then altered mental status is assumed if GCS<15 or AVPU<A.
Score 1 point for low oxygen saturation if oxygen saturation is <94% or measured on supplemental oxygen.

Missing data:
Assume any missing variable scores zero

Suffoletto strategy

Dichotomous assessment, positive if any of the following criteria are met:

- Systolic BP <100 mmHg
- History or suspicion of fever
- Prehospital judgment of infection

Modification:
Infer history or suspicion of fever from temperature>38

Missing data:
Assume any missing criterion is negative

UK Sepsis Trust Red Flags

Any of the following gives a positive score:

- Objective evidence of new or altered mental state
- Systolic BP ≤ 90 mmHg (or drop of >40 from normal)
- Heart rate ≥ 130 per minute
- Respiratory rate ≥ 25 per minute
- Needs O2 to keep SpO2 ≥ 92% (88% in COPD)
- Non-blanching rash / mottled / ashen / cyanotic
- Lactate ≥ 2 mmol/l
- Recent chemotherapy
- Not passed urine in 18 hours

Modification:
New or altered mental state assumed if the GCS verbal scale is <5. If the GCS verbal scale is missing, then new or altered mental state is assumed if GCS<15 or AVPU<A.
Drop BP change from normal and just use systolic BP ≤ 90 mmHg
Simplify O2 criteria to SpO2 < 92% or measured on supplemental oxygen
Drop non-blanching rash / mottled / ashen / cyanotic
Drop lactate ≥ 2 mmol/l
Drop recent chemotherapy
Drop not passed urine

Missing data:
Assume any missing criterion is negative.
